# Identification and Purification of Potential Bioactive Peptide of *Moringa oleifera* Seed Extracts

**DOI:** 10.3390/plants9111445

**Published:** 2020-10-27

**Authors:** Sangeeta Chandrashekar, Raman Vijayakumar, Ramachandran Chelliah, Deog-Hwan Oh

**Affiliations:** 1 Department of Food Science and Biotechnology, College of Agriculture and Life Science, Kangwon National University, Chuncheon 24341, Korea; gsangeetakv@gmail.com; 2 Department of Physiology, Bharath Institute of Higher Education and Research, Chennai 600 073, India

**Keywords:** *Moringa oleifera*, seed extract, bioactive peptide, cell aggregation, growth inhibition, clinical pathogens

## Abstract

The aim of the study was to investigate the antibacterial and anticoagulant activity of Moringa (*Moringa oleifera*) seed extracts and coagulant protein for their potential application in water treatment. Pathogenic microorganisms were obtained from Ramachandra Hospital, Chennai, India. Bacterial cell aggregation and growth kinetics studies were employed for six bacterial strains with different concentrations of seed extracts and coagulant protein. Moringa seed extract and coagulant protein showed cell aggregation against six bacterial strains, whereas seed extract alone showed growth inhibition of all six bacterial strains for up to 6 h, compared to that of control. *Escherichia coli* and *Salmonella para typhi* B did not develop resistance against coagulant protein. The results imply that *Moringa oleifera* is likely an efficient low-molecular bioactive peptide (with <7.5 kDa plant-based coagulant and antimicrobial peptides, confirmed by applying amino acid sequences), using liquid chromatography–mass spectrometry and HPLC, with the corresponding sequences from Napin-1A peptide posing different degrees of antibacterial activity against different pathogenic organisms.

## 1. Introduction

A major problem is poor water quality, and it is estimated that waterborne diseases affect about 37.7 million Indians annually; 1.5 million children under the age of five die due to diarrhea each year [1]. Microbial contamination through fecal contamination in water is the major reason for the poor water quality in India, and other developing countries, transmitting a large number of diseases. The common pathogens present in the drinking water include *Shigella* species, *Salmonella* species, *Klebsiella* species, *Escherichia coli*, *Enterobacter* species, and parasites such as *Giardia lamblia* and *Entamaeba histolytica* [2]. The available water treatment processes are expensive, especially in the developing countries. Synthetic organic and inorganic chemicals are commonly used for various water treatment processes, and are associated with environment and human health problems [3].

As an alternative, natural materials (plant material) can be used for water treatment. *Moringa* (*Moringa oleifera*) is one of the widely cultivated species in tropical regions of Asia, Africa, and South America, and it is used in rural areas of Africa for water treatment [4]. Previous study indicates that various parts of Moringa, such as leaves, roots, and bark, have antibacterial activity, and the seeds are well known for coagulant and antibacterial properties [5]. The seed extract and recombinant protein of *M. oleifera* is effective against *Staphylococcus aureus*, *Streptococcus pyrogenes*, *Streptococcus mitis*, *Streptococcus pneumoniae*, *Enterococcus faecalis*, *Escherichia coli*, and *Legionella pneumophila* for up to 150 min [6]. Though *Moringa* seed extract has been extensively studied to be a potent coagulant and antimicrobial agent, there are some concerns, such as i. the availability of Moringa seeds and cost; ii. difference in coagulation property of seed extracts collected from different localities; and iii. the type and quality of surface water (presence of contaminants, physical, and chemical properties) to be treated. A large screening study was conducted in order to find coagulant protein from plant materials as a complement to *Moringa* seeds for water treatment in Southern India.

**Figure 1:**
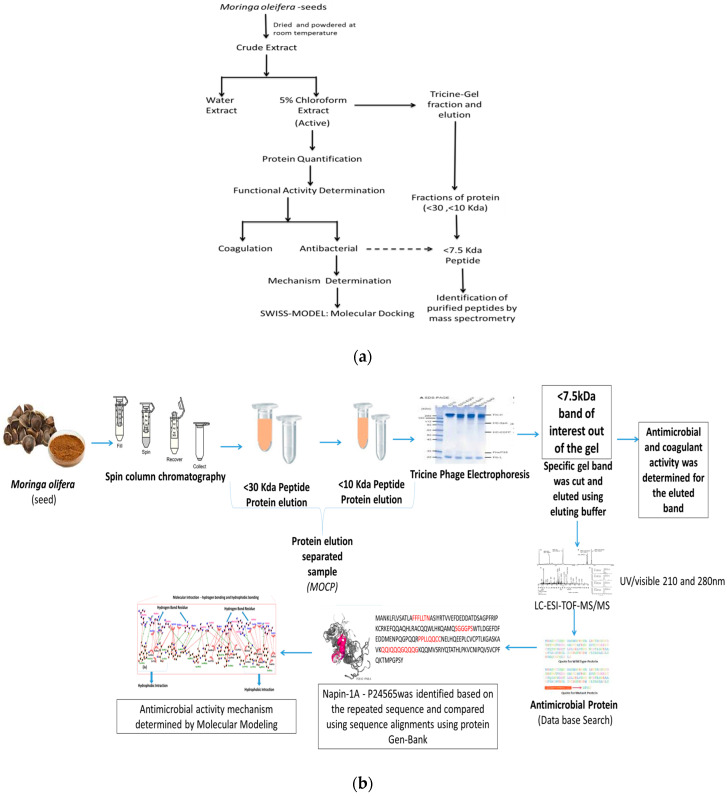
(**a**) Bioactivity-guided isolation of the antimicrobial compounds from *Moringa* seeds. (**b**) Outline on identification and purification of bioactive peptide from *Moringa* seeds.

The aim of this study was to investigate the antibacterial effect of *Moringa* seed extracts and coagulant protein against thirteen different clinical pathogens isolated from patient samples in India (Figure 1a). The *Moringa* seeds were collected in Tamilnadu, Southern India; the coagulant protein was separated by ion exchange chromatography; the effect of crude extract and coagulant protein on bacterial cell aggregation was performed by microscopic observation; and growth kinetics was performed by turbidity measurement in a spectrophotometer. The antibacterial activity of the coagulant proteins and their potential water treatment agent is discussed.

## 2. Materials and Methods

### 2.1. Extraction and Purification of Coagulant Protein

#### Collection and Preparation of Crude Extract from Plant Seeds

*Moringa* (*Moringa oleifera*, MO) seeds were purchased from local shops in Southern India. Preparations of crude extracts from *Moringa* seeds were performed as described earlier [7]. Seed coats (husks) were removed from MO seeds, and the seed kernels were grounded into fine powder by using a mortar and pestle. In order to remove oil from the fine powder, 90% ethanol was added, and the supernatant was separated by centrifugation at 4000 rpm for 15 min. The pellet was allowed to air dry, and 5% chloroform extract was prepared using chloroform and sterile distilled water. The obtained soluble fraction is referred to as crude extract (CE) (Figure 1a).

### 2.2. Identification and Purification Bioactive Peptide

#### 2.2.1. Purification of Coagulant Protein

The CEs were further purified by spin column chromatography (Pall Corporation Macrosep Advance Centrifugal Device, New York, NY, USA) as described by Chelliah et al. [8], in a batch system. The cell free supernatant (CFS) was filtered in the <30 kDa spin column, and centrifuged at 3000 rpm for 30 min. The <30 kDa filtrates were transmitted to a <10 kDa spin column, and centrifuged at 4000 rpm for 30 min. Finally, the filtrate was collected and tested for coagulation and antimicrobial activity by applying the disc diffusion method, through which partially purified low-molecular-weight peptides were tested against *Escherichia coli* 0157:H7, *Salmonella paratyphi B*, *Salmonella typhimurium*, *Shigella flexneri*, *Salmonella paratyphi A*, and *Kelbsiella pneumoniae*. The molecular weight of coagulation and antimicrobial peptide falls under 7.5–6 kDa (Figure 1b).

#### 2.2.2. Protein Determination

The protein content of MO crude extracts and coagulant protein was estimated by Lowry’s method [9]. The samples were prepared based on denaturation of protein (by heating in the presence of a sample buffer containing 1% Tricine buffer with or without a reducing agent, such as 20 mM Dithiothreitol (DTT), 2-mercaptoethanol (BME) or Tris(2-carboxyethyl)phosphine (TCEP)). Lysate protein was precipitated overnight at −20 °C, and centrifuged at 4000 rpm with acetone 1:5 (*v*/*v*) at 4 °C for 10 min. The purity of the coagulant protein was analyzed by Tricine gel analysis according to Laemmli [10], with buffers according to Fling and Gregerson [11]. Novex Tricine (EC6 675BOXNovex™ 10%, Tricine, 1.0 mm, Mini Protein Gel, 10-well) Protein Gels provide separation of low molecular weight proteins and peptides, which are applied at a low pH level in the gel buffer (LC3675 Novex™ Tris-Glycine Transfer Buffer (25X)) and substituted to Tricine for glycine in the running buffer. The smaller proteins and peptides that migrate with the stacked dodecylsulfate (DS) ions in the Tris-glycine gel system are well separated from DS ions in the Novex Tricine Gel System, offering sharper bands and higher resolution. The gels were stained with Coomassie brilliant blue to visualize the protein; the gels were further washed with sterile distilled water three times, and a <7.5 kDa band of interest taken out of the gel with a blade, with excessive gel trimmed away. Excessive liquid associated with the gel was removed by touching with filter paper. Then 1–2 excised gel pieces were placed at the bottom of a 200 μL tube provided (the tube can accommodate multiple pieces of excised gels).

#### 2.2.3. Tricine-Gel Fraction and Elution

Protein encoded bands were set in 5% (*v*/*v*) glutaraldehyde for 25 min. The low molecular weight 7.5 KD1a band was cut into pieces of gel, using the flat end of the pestle, and extraction powder (about 1/4 to 1/3 the volume of the gel) was added to the gel at bottom of the tube. Elution buffer (0.1–0.5% SDS or acid labile surfactants) was added to the tube (20 μL/piece of gel), and protein pellets (100 μg) were re-suspended into a buffer loading 25 μL (4% SDS, 20% glycerol, 10% 2-mercaptoethanol, 0.004% Bromophenol blue, and 0.125M Tris/HCl, pH 6.8), and incubated for 5 min at 90 °C.

#### 2.2.4. Identification of Purified Peptides by Mass Spectrometry

Sequential identification of peptides by mass spectrometry–liquid chromatography–electrospray ionization–quantitative time-of-flight tandem–mass spectrometry (LC–ESI–TOF–MS–MS) were analyzed at the National Instrumentation Center for Environmental Management of Seoul National University in Korea, according to an earlier method by Chelliah et al. [8]. Analyses were performed through applying high-performance liquid chromatography (HPLC) (UltiMate3000 Series, Thermo Fisher Scientific, Waltham, MA, USA), a combined arrangement encompassing a self-regulative nano pump, a self-sampler (MDS SCIEX, Seoul, Korea), and an integrated hybrid quadrupole-time-of-flight (TOF) mass spectrometer (Applied Biosystems, Seoul, Korea). The samples were ionized using nano-electrospray ionization. Further 1.5 g of the spin column eluted peptide was dissociated in 50 mL of sterile distilled water. Diverse elutes of fractions (1.5 µL) of the sample were inserted in LC-nano ESI–MS–MS. The samples were entombed in a ZORBAX 300SB-C18 ruse column (5 µm particle size, 300 µm i.d. × 5 mm, 100 pore size) (Agilent Tech, Santa Clara, CA, USA) and washed for 6 min, graded with solvent-A (water/acetonitrile (98:2, *v*/*v*), 0.1% formic acid) 98% and solvent-B (water/acetonitrile (2:98, *v*/*v*) 2% and 0.1% formic acid) at a flow rate of 5 µL/min. The peptides were segregated on a capillary column (75-µm i.d. × 150 mm, 3.5 µm subdivision size, 100 pore size, part number 5065-9911) (Zorbax 300SB-C18) at a transfer rate of 290 nL/min with a gradient at 2–35% solvent-B over 30 min, then from 35–90% over 10 min, followed by 90% solvent-B for 5 min, and, finally, 5% solvent-B for 15 min. The electrosprays were smeared by 2250 eV, based on a coated silica tip. The peptides were inspected using QS 3.0 software (Applied Biosystems, Seoul, Korea). The ranges of proteins were identified based on 300–3000 *m*/*z* values.

#### 2.2.5. SWISS-MODEL: Homology Modeling of Protein Structures

The protein models were spawned by a structural bioinformatics web-server dedicated to homology modeling of 3D protein structures (SWISS-MODEL), licensed under the Creative Commons Attribution-Share Alike 4.0 (CC BY-SA 4.0) International License, based on the sequence generated by liquid chromatography–mass spectrometry (LC–MS). SWISS-MODEL generates theoretical models by automated homology modeling techniques developed by the Computational Structural Biology Group at the Swiss Institute of Bioinformatics (SIB) at the Biozentrum, University of Basel, Switzerland.

### 2.3. Functional Activity of Bioactive Peptide

#### 2.3.1. Coagulation Activity

The crude extracts and the coagulant proteins were tested for coagulation activity, using synthetic clay solution as described earlier by Sami [7]. Clay solution (1%) was prepared using kaolin clay (using 1 or 2 L volume beakers fitted with mechanical stirrers, the optimum coagulant dose is generally calculated from jar test analysis) and mixed with protein to get a final volume of 1 mL and initial optical density at 500 nm, and, after 60 min, was measured (this approach not only eliminates the requirements for clay suspension sample volume and coagulant dosage, but also makes it possible to analyze large numbers of samples simultaneously). By constantly recording optical density (OD500), it is ideal to quickly screen out active and non-active coagulants, and to observe settling characteristics of the flocks. Coagulant solutions (10 mL) in the 1 mL cuvette were applied to the high-turbidity 1 mL clay suspension (250–300 NTU), and immediately homogenized. Using a UV–visible spectrophotometer (Cary60 Bio), this was allowed to settle for 1 h, and absorbance was measured at 500 nm. In order to minimize the background effect, a sample volume of 200 mL from the top was transferred for absorbance measurements to a quartz glass cuvette (type 105.200-QS, 10 mm light direction, HELLMA) (the decrease in absorbance, relative to control, determines the activity of coagulation). The percentage of coagulation activity was calculated using the formula: coagulation activity % = ((initial absorbance − final absorbance)/initial absorbance) × 100.

#### 2.3.2. Antibacterial Activity

##### Microbial Strains

Clinical isolates of thirteen bacterial strains: Escherichia coli, Salmonella paratyphi B, Salmonella typhimurium, Shigella flexneri, Salmonella paratyphi A, and Klebsiella pneumoniae were obtained from the Department of Microbiology, Ramachandra University, Chennai, India. Stock culture was maintained in nutrient agar media at 4 °C.

##### Bacterial Cell Aggregation

The clinical isolates were grown in nutrient broth (NB) and incubated at 37 °C in a shaking incubator overnight, and diluted to an initial optical density (OD) of 0.1 and 0.3 at 600 nm for growth kinetics or cell aggregation test, respectively. The concentrations of the protein that was used for cell aggregation tests were: *Moringa* CE 0.041, 0.020, 0.012, and 0.008 mg/mL; *Moringa* coagulant purified protein (CP) 0.006, 0.012, 0.015, and 0.03 mg/mL. CE and CP were added to the culture suspension with an initial OD of around 0.3, and incubated at 37 °C for 4 h. The cell aggregation was observed at each hour under phase-contract microscope (Nikon Eclipse 80i, National High Magnetic Field Laboratory (NHMFL), The Florida State University, Tallahassee, Japan), and the images were captured using a microscopic digital camera (EM-200F, USB-2.0, CCD Chip).

##### Determination of Minimum Inhibitory Concentration (MIC), Minimum Bactericidal Concentration (MBC), and Growth Kinetics

The growth kinetics studies were conducted by adding different concentrations of protein to pre-diluted overnight cultures (0.1 OD) to a final volume of 1 mL in a sterile cuvette, and incubating at 37 °C for 4–6 h, with continuous shaking. The OD at 600 nm was measured every 60 min by spectrophotometer (Biophotometer, Eppendorf Korea., Gangnam-gu Seoul, Korea), and values were recorded.

##### Determining the Purified Antimicrobial Peptides Molecular Mechanism

In Silico Molecular Interaction Analysis and Docking for Antimicrobial Peptides in *Moringa oleifera* Seed Extract (MOS)

In silico molecular docking was performed to assess the docking ability of Napin with Lipid II, 4PLB, and LipoXc. The 3D structure of napin, Lipid II, 4PLB, and LipoXc was downloaded from Research Collaboratory for Structural Bioinformatics-Protein Data Bank (RCSB-PDB). Proteins were prepared before docking, and provided to Cluspro (v2.0) [12]. The Cluspro web server is a widely-used tool for protein–protein docking. It works on a fast Fourier transform correlation approach, where simple scoring functions evaluate docking confirmation. Cluspro performs multi-stage protocol: rigid body docking, energy-based filtering, evaluating structures based on clustering properties, and, finally, returning a small number of structures based on minimized energy. The server returns models based on energy and cluster size, and amongst all, one of the returned models was selected, based on the lowest energy and size of the cluster.

Binding energy surface properties were calculated, which in turn gives the protein interface probability and the interaction site of two proteins. For surface properties calculation, Surface Racer 5.0 was used, which calculates exact accessible surface area (ASA), molecular surface area (MSA), and cavities to the inner protein inaccessible to solvent from outside. The output includes surface area of the docked proteins models for each residue, in addition to those of individual atoms [13].

## 3. Results and Discussion

### 3.1. Purification Bioactive Protein and Quantification of Total Protein

#### 3.1.1. Purification of Coagulant Protein

**Figure 2:**
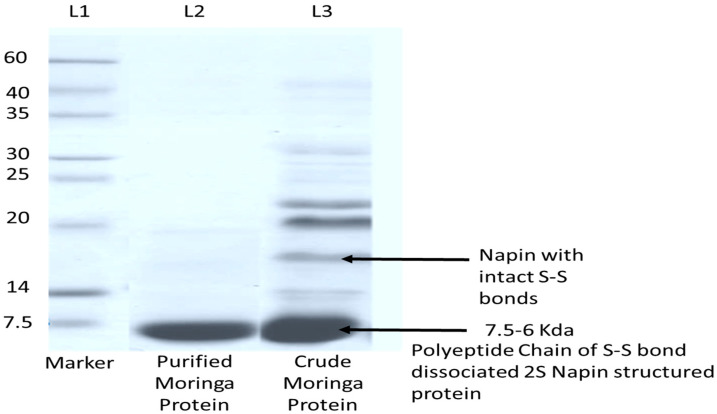
Polypeptide profiles of *Moringa* extract in 8–25% gradient precast gel. MWM: molecular weight markers. Coagulation and antimicrobial activity of *Moringa* crude extract (CE) and purified protein (CP) (spin column chromatography < 10 kDa). Lane 1: Marker Sigma-Aldrich (7.5–6.0 kDa); Lane 2: Purified *Moringa* (<7.5 kDa); Lane 3: Crude *Moringa* protein extract.

The protein concentration was found to be higher in crude extract from MO seed extract. The coagulant protein was separated from the crude seed extracts of MO by spin column chromatography (SCC). The eluted protein fraction was concentrated, and showed similar coagulation activity as that of crude extract. The molecular mass of coagulant protein was determined by SDS-PAGE (Figure 2). The coagulant protein was separated after SCC of MO showed a protein band of less than 7.5 kDa. These results were strongly correlated with earlier reports on coagulant protein from MO [14].

#### 3.1.2. Identification of Purified Peptides by Mass Spectrometry

**Figure 3:**
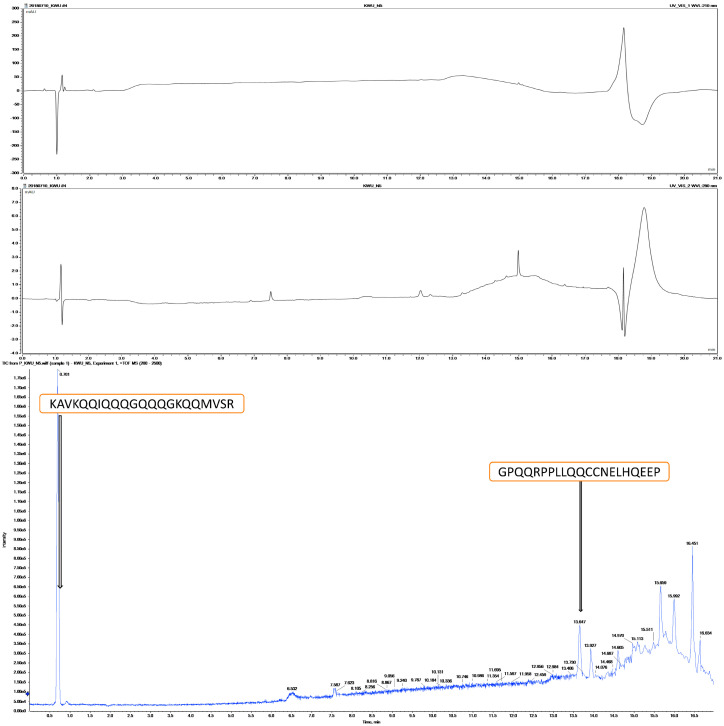
Based on the spin column chromatography, a chromatogram of <7.5 kDa filter and Tricine-gel eluted peptide fraction, on sequential identification of peptides by mass spectrometry–liquid chromatography–electrospray ionization–quantitative time-of-flight tandem–mass spectrometry (LC–ESI–TOF–MS–MS) UV–visible 210 and 280 nm.

**Figure 4:**
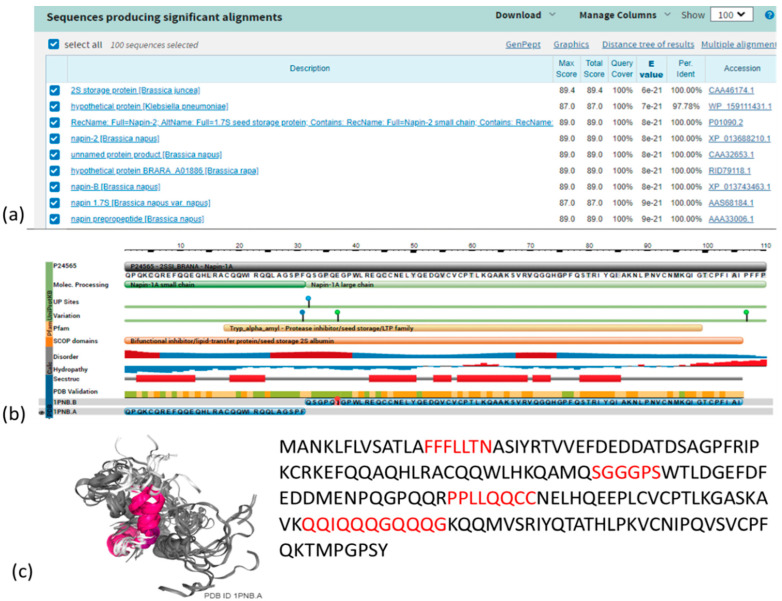
(**a**) Based on liquid chromatography–mass spectrometry (LC–MS) data, <7.5 kDa peptide was identified based on the repeated sequence, and compared using sequence alignments using protein Gen-Bank coding sequence (CDS) translations + PDB + Swiss-Prot + PIR+PRF, excluding environmental samples from whole genome sequence (WGS) projects. (**b**) The sequence similarity of <7.5 kDa peptide was identified and compared, and found as Napin-1A. (**c**) Subunit structure: The mature protein consists of a small and a large chain linked by disulfide bonds -Napin-1A-P24565 (2SSI_BRANA), Biological Macromolecular Structures Enabling Breakthroughs in Research and Education, https://www.rcsb.org/pdb/protein/P24565?addPDB=1PNB#.

The specific < 7.5 kDa peptide band from the Tricine-gel was eluted using elution buffer (0.1–0.5% SDS or acid labile surfactants) to the tube (20 μL/piece of gel); further, the eluted bands from <7.5 kDa peptides were identified in the low molecular weight peptide profile, and plant protein profiling is displayed in Figure 3. A total of 2173 peptides were notorious and eluted, and further tested in vitro for antimicrobial activity; the peptide sequences were screened using an in silico platform for pediocin protein and peptide profiling, and developed QS 3.0 software (Applied Biosystems, Seoul, Korea) was used to predict potential antimicrobial peptides (AMP). Although many potential antimicrobial inhibitory peptides were identified, peptides FFFLLTN, SGGGPS, PPLLQQCCNEL, KAVKQQIQQQGQQQGKQQMVSR, and GPQQRPPLLQQCCNELHQEEP (Figure 4) (Supplementary Table S1) were most abundant, which showed the strongest antimicrobial activity (0.207 µg) of <7.5 kDa fraction [8,15,16]. As *Moringa* coagulant protein has been reported as a complement to the chemicals in drinking water treatment process, the present study reveals that the antimicrobial property of *Moringa* extract provides a good substitute to treat water, thereby reducing the risk of infection caused by water contamination. 

### 3.2. Determination on Functional Activity of Bioactive Proteins

#### 3.2.1. Coagulation Activity

**Figure 5:**
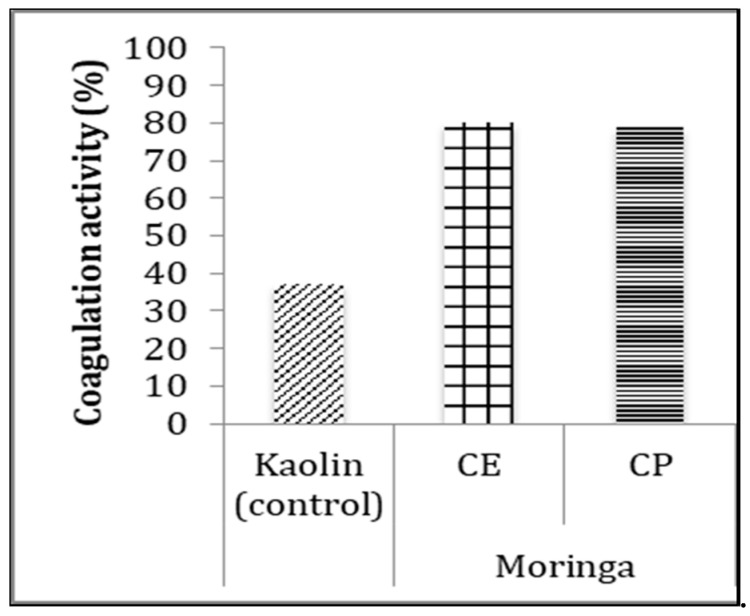
Coagulation activity of *Moringa* (MO) crude extract (CE) and <7.5 kDa purified protein (CP) after 90 min of sedimentation. The CEs were not diluted, whereas CP was five-times diluted. Kaolin clay without addition of extract served as a control. Results were presented significantly at different values (*p* < 0.05).

The coagulation activity of *Moringa* (MO) crude seed extracts and coagulant protein were measured using kaolin clay solution, and clay solution alone used as a control (Figure 5).

#### 3.2.2. Antibacterial Activity

The antibacterial activity of *Moringa* seed extract and coagulant protein was analyzed against six clinical pathogenic strains.

##### Cell Aggregation Activity

**Figure 6:**
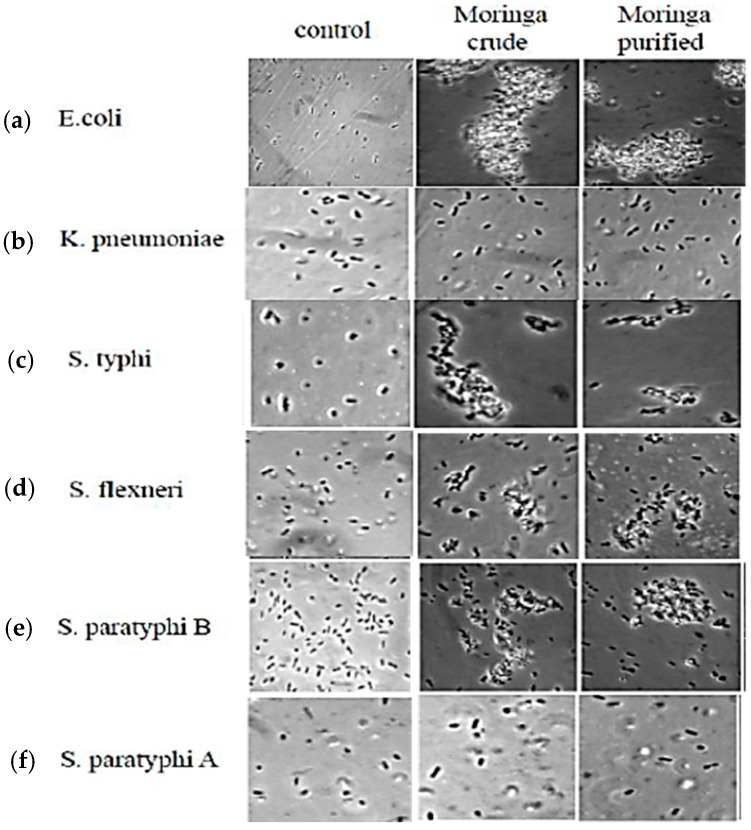
Comparative analysis for aggregation experiments of *Moringa* seed extracts towards six different clinical pathogens: (**a**) *Escherichia coli*, (**b**) *Klebsiella pneumonia*, (**c**) *Salmonella typhi*, (**d**) *Shigella flexneri*, (**e**) *Salmonella paratyphi A*, and (**f**) *Salmonella paratyphi A* (control: untreated sample).

Clinically-isolated dysentery causing enteropathogens was tested for cell aggregation by MO crude extracts and coagulant protein. All the six bacterial strains were isolated from patient samples and identified at Ramachandra University, India. Among thirteen different strains tested, MO crude extract (CE) and coagulant purified protein (CP) showed cell aggregation of six bacterial strains. The results from the aggregation experiments are shown in Figure 6. Both CEs were effective after 2 h of incubation against larger flocs of bacterial cells of *Escherichia coli 0157:H7*, *Salmonella paratyphi B*, *Salmonella typhi*, *Shigella flexneri*, *Salmonella paratyphi A*, and *Kelbsiella pneumoniae*, compared to that of *Moringa* CE. The possible reason could be that more than one seed storage protein that has coagulation properties could play a role in aggregation of bacterial cells, as it was observed that the eluted fraction from ion exchange matrix revealed protein bands having molecular mass of 7.5 kDa. Aluko [17] reported that the peptide sequence of 9 kDa protein from Mus seed was similar to Napin-3 protein, which has sequence homolog to coagulant protein. Further study is needed to understand the mechanism of action against bacterial strains.

The key structural features of *Moringa olifera* < 7.5 kDa purified protein (MOCP) are attributes of its functionality as an antimicrobial agent. MOCP is a cationic protein, and contains a net positive charge that facilitates the interaction with microbial anionic lipid membranes. MOCP also possesses an amphiphilic helix–loop–helix motif that helps it to integrate into the bacterial membranes. Owing to these functional properties, MOCPs are selectively targeted, and kill several microbes, including waterborne pathogens. It has been established that membrane fusion is the most dominant mechanism of MOCP antimicrobial activity [17]. The flocculation of the negatively-charged particles (impurities) is a result of the binding of positively-charged macromolecules (cationic polymer) to the surfaces of particles by Coulomb forces. The neutralization of part of the surface charge and reduction in the electrostatic repulsion leads to agglomeration of particles. Meanwhile, only a small part of the charged macromolecule binds to the surface of one particle, even as the major portion is free to bind to the surface of another particle. This leads to agglomeration and formation of flocs by bridging between negatively-charged particles. The path ‘patch charge’ mechanism varies, based on the nature of MOCP and different types of pathogens.

##### Determination of Minimum Inhibitory Concentration (MIC) and Minimum Bactericidal Concentration (MBC) Antimicrobial Activity of Peptide on Bacterial Growth Kinetics

**Figure 7:**
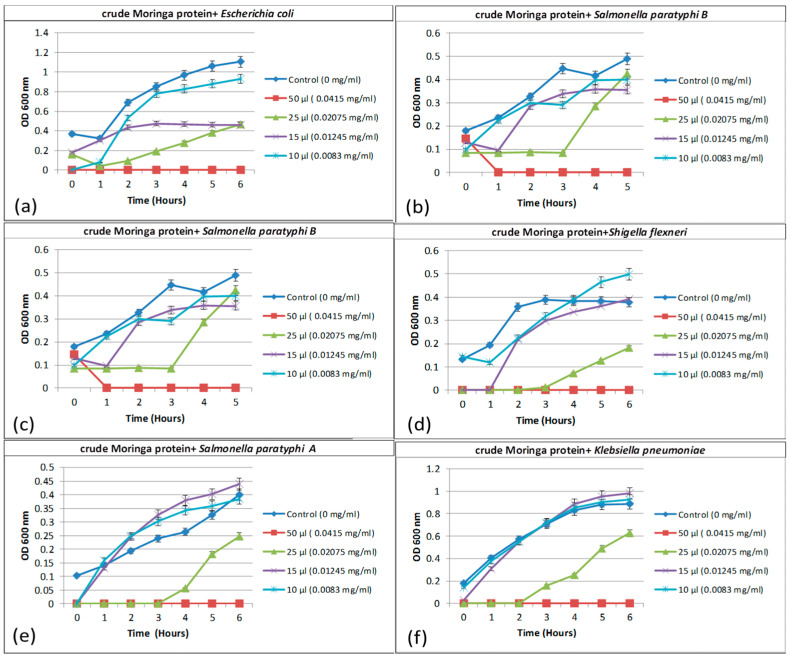
Growth kinetics of different concentration of *Moringa* crude extract against clinical pathogens (**a**) *Escherichia coli*, (**b**) *Salmonella paratyphi B*, (**c**) *Salmonella typhimurium*, (**d**) *Shigella flexneri*, (**e**) *Salmonella paratyphi A*, and (**f**) *Klebsiella pneumoniae* (control: untreated sample). Results were presented significantly at different values (*p* < 0.05).

The growth kinetics experiments were performed on all six clinical isolates with CE and CP from *Moringa* seed (Figure 7). The crude extract (CE) of *Moringa* seed showed growth inhibition up to 6 h against *Escherichia coli 0157:H7, Salmonella paratyphi B*, *Salmonella typhimurium*, *Shigella flexneri*, *Salmonella paratyphi A*, and *Kelbsiella pneumoniae*. The concentration of CE ranging between 0.041–0.008 mg/mL is required for the complete inhibition of growth. Among the isolates tested, *Salmonella typhimurium* affected by 0.02 mg/mL of MC was effective, and showed growth inhibition up to 3 h; thereafter, it started to grow slowly. On the other hand, the optical density was lower, compared to that of the control. The reduced growth rate mentioned in Figure 4 refers to inhibition of the growth up to 3 h. Broin [18] reported that the minimal inhibitory concentration (MIC) for *E. coli* was 50 mg/mL up to 150 min. In this study, the CE of *Moringa* seed showed growth inhibition at low concentration of 0.008 mg/mL up to 6 h, proving the efficiency of the proteins present in the CE. The possible reason could be the extraction method used to get soluble proteins from the crude extract and/or the variety of the seed used in this study and the efficiency of coagulant protein. This is very well in correlation with earlier reports that stated that when drinking water is treated with crude seed extract of *Moringa* and stored for a longer time, it will change taste and odor, due to organic load and disinfection byproducts [19,20]. Therefore, it is essential to separate the coagulant protein from the crude extract, and test for antibacterial activity and water treatment.

The growth kinetic experiment was performed against six different clinical pathogens with MO coagulant. The isolates were selected based on the cell aggregation and effect against CE. All the strains tested showed reduced growth rate at the concentration of 0.02 mg/mL (Table 1). In comparison, the CP alone was not as effective as that of CE. Earlier reports on *Moringa* CP showed growth inhibition against *E. coli* [20]. The reason for the differences in the behavior could be that different bacterial isolates were selected for antibacterial test of CP; for instance, Saleem et al. [8] reported using *E.coli* D31 and *Bacillus thuringiencis* strain Bt7, and showed growth inhibition up to 6 h. In the present study, the pathogenic organisms tested for antibacterial activity were isolated from patients. The other possible reason could be the variety of *Moringa* seeds used for isolating the coagulant protein. In the present study, the *Moringa* seed was collected in Southern India, whereas the seeds collected from different places, such as Kenya and other geographical regions, were reported. Further study is needed to explore the differences in coagulant protein from different localities and varieties of *Moringa* seed by protein sequence comparison and mechanism of action against different pathogenic strains. To our knowledge, this is the first report on *Moringa* (<7.5 kDa) seed protein showing bacterial cell aggregation and reduction in growth rate against clinical isolates.

##### Molecular Interaction of AMP with Pathogens (Mode of Action)

SWISS-MODEL: Homology Modeling of Protein Structures

Molecular dynamics were considered to be an efficient tool to form a simulation model in order to find linkage between plant-based bioactive (napin peptides; small molecular size proteins which are produced as a secondary metabolite) compounds, in addition to their respective targets, which enable interactions at specific time period. The three-dimensional quantitative modeling (3D-QSAR), to understand the relationship of specific amino acids of p Progress in 3D quantitative structural activity relationship modeling (3D-QSAR), has reopened a method to compare the plant secondary metabolites and their targeted molecular function. The three-dimensional structure of napin peptide analog 5 (Supplementary Figure S1) is the structure-made sequence which is based on similar proline and glutamine-rich peptide motifs. Using Discovery Studio 4.5 Visualizer [21] and Chimera 1.9, the docked structures were investigated [22,23].

According to the in silico molecular modeling docking analysis, the first loop region of napin was formed by three different amino acids, such as isoleucine (Ile), serine (Ser), and proline (Pro), between the 18th and 20th positions, whereas the secondary loop, based on glycine (Gly), glutamine (Gln), and valine (Val), was between the 39th to 41st positions. The secondary structure of napin peptide is the electrostatic potential of the protein with various ligands. The electrostatic potential was calculated using a Poisson–Boltzmann equation-based algorithm (Supplementary Figure S1a–c). The blue color represents positive domains of electrostatic potential of 3.0 kcal/mole (total charge of +5); however, the electrostatic potential negative domains are represented in red, and documented in three-dimensional structure, showing the ambiguous comportment of positively-charged protein networking electrostatic interaction between the negative domains and positively charged ions (Supplementary Figure S1). SPRINT, a compendium of diagnostic protein family fingerprints, was researched to classify sequence similarities to recognized antimicrobial peptides to check for antimicrobial sequence motifs within the napin sequences [23]. The results showed that napins are categorized within their corresponding sequences, with some recognized antibacterial peptides, and have similar antimicrobial sequence motifs. A search query in one of the antimicrobial peptide databases, such as APD (http:/aps.unmc.edu/AP/database/mysql.php) and AMPed (https:/amped.uri.edu/index.php), is one of the most straightforward methods to verify whether a query protein has been identified as an antimicrobial peptide, based on experimental proof.

The results show that the proteins of both napins have a high binding affinity to selective bacterial enzymes, implying that they can inhibit bacterial activity. The lowest value represents the strongest binding force, as far as global force is concerned [24]. It can be concluded, on the basis of this, that napin directly binds to the 50S ribosomal subunit of *Deinococcus radiodurans* (PDB ID: 1XBP) and *Staphylococcus aureus* gyrase B (PDB ID 4URM). Conversely, procruciferin that binds to dihydrofolate reductase (PDB ID 3FYV) and *S. aureus* is found to be better. Therefore, the molecular docking analysis shows napin as an antimicrobial agent. Trimetrexate, pyrimethamine, methotrexate, and trimethoprim, well-established drug molecules, have been reported to bind with 3FRA [25]. The findings also show that both the napin proteins bind by hydrogen bonding with the 50S ribosomal subunit of *D. radiodurans* (1XBP). Novel antibacterial compounds with substituted amino moieties derived from pleuromutilin have been reported to exert antibacterial activity by binding to 1XBP [24].

Some previous simulation work inside a model bacterial membrane environment on lipid-II and antimicrobials has been completed. The identification of lipid-II by vancomycin and vancomycin was modeled by Jia and colleagues [25]. Then came the dimerization, using the GROMOS 54A7 force field, of these complexes. Using Molecular dynamics (MD) simulations, Chugunov et al. [26] conducted a comprehensive study of the behavior of lipid-II in a membrane, finding different lipid-II tail conformations in the bilayer and an induced amphiphilic “landing terrain” pattern on a model bacterial membrane surface not seen in a reference membrane. Subsequently, MD simulations are used to integrate conformational flexibility into both complex partners and allow us to determine whether key interactions continue, in the sense of full atomistic molecular mechanics. A docking site for a second lipid II nisin molecule has not yet been identified and published in the literature, nor has the pore formation mechanism been described. A binding site at the C-terminus of D-Ala5 of lipid II is proposed in the present work, and shows that this 2:1 complex is stable in MD simulations.

## 4. Conclusions

The effects of crude extract and coagulant protein from *Moringa* seeds were investigated against six different pathogenic microorganisms. It is evident that crude extract and coagulant protein from *Moringa* seed protein has antibacterial activity when measured by cell aggregation and growth inhibition studies. Furthermore, based on mass spectrometry–liquid chromatography–electrospray ionization–quantitative time-of-flight tandem–mass spectrometry (LC–ESI–TOF–MS–MS), it was found that <7.5 kDa peptide of *Moringa* seed extracts could effectively have antibacterial activity. Furthermore, the elucidation of the mechanisms behind such aggregation and inhibition activities would widen the understanding of such mechanisms, and thus help to identify such similar coagulant molecules that can be effectively used in the water treatment process.

## Figures and Tables

**Table 1:** Determination on level of aggregation (Mild (+), High (++), No aggregation (-)), minimal inhibitory concentration (MIC), and minimal bactericidal concentration (MBC) with growth kinetics.

S.No	Food Bone Pathogens	Bacterial Cell Aggregation		Moringa Olifera Crude Extract (MOCE)	
		Level of Aggregation (MOCE)	Level of Aggregation (MOCP)	Minimal Inhibitory Concentration (MIC) (mg/mL)	Minimal Bactericidal Concentration (MBC) (mg/mL)
*1*	*Escherichia coli 0157:H7*	++	++	0.0124	0.0415
*2*	*Salmonella paratyphi B*	++	++	0.0207	0.0415
*3*	*Salmonella typhi*	++	++	0.0207	0.0415
*4*	*Shigella flexneri*	++	++	0.0207	0.0415
*5*	*Salmonella paratyphi A*	+	+	0.0207	0.0415
*6*	*Kelbsiella pneumoniae*	Mild	Mild	0.0207	0.0415
*7*	*Media*	-	-	-	-

## Supplementary Materials

The following are available online at https://www.mdpi.com/2223-7747/9/11/1445/s1. Table S1: LC-ESI-TOF-MS/MS analysis of Moringa olifera (MOP)-derived peptides (≤7.5 kDa) after purification based on spin coloum chromatography. Figure S1. (a) (i) The interaction analysis between napin (pink) and lipid 2 (blue) by molecular docking; (ii) the interaction model of complex napin and lipid 2−3D structure of protein complex; (iii) interfaces and key residues analysis; (iv) Ligplot representation showing hydrogen and hydrophobic interactions between pediocin and lipid 2 complex and surface area assessment table for best docked models.

## References

[1] Khan F.M., Gupta R. (2020). Escherichia coli (*E. coli*) as an Indicator of Fecal Contamination in Groundwater: A Review. International Conference on Sustainable Development of Water and Environment.

[2] Bain R., Cronk R., Wright J., Yang H., Slaymaker T., Bartram J. (2014). Fecal contamination of drinking-water in low-and middle-income countries: A systematic review and meta-analysis. PLoS Med..

[3] Hopkins K.G., Noe G.B., Franco F., Pindilli E.J., Gordon S., Metes M.J., Claggett P.R., Gellis A.C., Hupp C.R., Hogan D.M. (2018). A method to quantify and value floodplain sediment and nutrient retention ecosystem services. J. Environ. Manag..

[4] Sato T., Qadir M., Yamamoto S., Endo T., Zahoor A. (2013). Global, regional, and country level need for data on wastewater generation, treatment, and use. Agric. Water Manag..

[5] Taiwo A.S., Adenike K., Aderonke O. (2020). Efficacy of a natural coagulant protein from Moringa oleifera (Lam) seeds in treatment of Opa reservoir water, Ile-Ife, Nigeria. Heliyon.

[6] Gupta S., Jain R., Kachhwaha S., Kothari S.L. (2018). Nutritional and medicinal applications of Moringa oleifera Lam—Review of current status and future possibilities. J. Herb. Med..

[7] Saleem M., Sami A.J., Bachmann R.T. (2020). Characterisation and coagulant activity screening of fractionated water-soluble seed proteins from Moringa oleifera. Mater. Today Proc..

[8] Chelliah R., Saravanakumar K., Daliri E.B.M., Kim J.H., Lee J.K., Jo H.Y., Kim S.H., Ramakrishnan S.R., Madar I.H., Wei S. (2020). Unveiling the potentials of bacteriocin (Pediocin L50) from Pediococcus acidilactici with antagonist spectrum in a Caenorhabditis elegans model. Int. J. Biol. Macromol..

[9] Protein det Lowry O.H., Rosebrough N.J., Farr A.L., Randall R.J. (1951). Protein measurement with the Folin phenol reagent. J. Biol. Chem..

[10] Schägger H. (2006). Tricine–sds-page. Nat. Protoc..

[11] Fling S.P., Gregerson D.S. (1986). Peptide and protein molecular mass determination by electrophoresis using a high-molarity tris buffer system without urea. Anal. Biochem..

[12] Kozakov D., Hall D.R., Xia B., Porter K.A., Padhorny D., Yueh C., Beglov D., Vajda S. (2017). The ClusPro web server for protein-protein docking. Nat. Protoc..

[13] Tsodikov O.V., Record M.T., Sergeev Y.V. (2002). A novel computer program for fast exact calculation of accessible and molecular surface areas and average surface curvature. J. Comput. Chem..

[14] Aluko R.E., McIntosh T., Katepa-Mupondwa F. (2005). Comparative study of the polypeptide profiles and functional properties of Sinapis alba and Brassica juncea seed meals and protein concentrates. J. Sci. Food Agric..

[15] Broin M., Santealla C., Cuine S., Kokou K., Peltier G., Joet T. (2002). Flocculant activity of a recombinant protein from Moringa oleifera Lam. Seeds. Appl. Microbiol. Biotechnol..

[16] Riaz M.B., Khan A.-U., Qazi N.G. (2019). Pharmacological and computational evaluation of fig for therapeutic potential in hyperactive gastrointestinal disorders. BMC Complement. Altern. Med..

[17] Attwood T.K., Bradley P., Flower D.R., Gaulton A., Maudling N., Mitchell A.L., Moulton G., Nordle A., Paine K., Taylor P. (2003). PRINTS and its automatic supplement, prePRINTSacid pharmacophores. Nucleic Acids Res..

[18] Silveira F.M.R., Baptista A.T.A., Dutra T.V., de Abreu Filho B.A., Gomes R.G., Bergamasco R. (2020). Application of Moringa oleifera Lam. fractionated proteins for inactivation of Escherichia coli from water. Water Sci. Technol..

[19] Pandey K.R., Naik S.R., Vakil B.V. (2015). Probiotics, prebiotics and synbiotics—A review. J. Food Sci. Technol..

[20] Bacha K., Tariku Y., Gebreyesus F., Zerihun S., Mohammed A., Weiland-Bräuer N., Schmitz R.A., Mulat M. (2016). Antimicrobial and anti-Quorum Sensing activities of selected medicinal plants of Ethiopia: Implication for development of potent antimicrobial agents. BMC Microbiol..

[21] Wang G., Li X., Wang Z. (2015). APD3: The antimicrobial peptide database as a tool for research and education. Nucleic Acids Res..

[22] Aamir M., Singh V.K., Dubey M.K., Meena M., Kashyap S.P., Katari S.K., Upadhyay R.S., Umamaheswari A., Singh S. (2018). In silico Prediction, Characterization, Molecular Docking, and Dynamic Studies on Fungal SDRs as Novel Targets for Searching Potential Fungicides Against Fusarium Wilt in Tomato. Front. Pharmacol..

[23] Schomburg K. (2014). Protein-Ligand Inverse Screening and Its Application in Biotechnology and Pharmacology. Ph.D. Thesis.

[24] Shang R., Wang S., Xu X., Yi Y., Guo W., Liang J. (2013). Chemical synthesis and biological activities of novel pleuromutilin derivatives with substituted amino moiety. PLoS ONE.

[25] Jia Z.G., O’Mara M.L., Zuegg J., Cooper M.A., Mark A.E. (2011). The Effect of Environment on the Recognition and Binding of Vancomycin to Native and Resistant Forms of Lipid II. Biophys. J..

[26] Chugunov A., Pyrkova D., Nolde D., Polyansky A., Pentkovsky V., Efremov R. (2013). Lipid-II forms potential “landing terrain” for lantibiotics in simulated bacterial membrane. Sci. Rep..

