# Supplementary material for: Identification and Purification of Potential Bioactive Peptide of Moringa oleifera Seed Extracts
Source: Plants (Basel). 2020 Oct 27;9(11):1445. doi: 10.3390/plants9111445 (PMC7716235; doi:10.3390/plants9111445)
Supplement: Supplementary file 1 [file plants-09-01445-s001.zip › plants-929926-supplementary/plants-929926-supplementary-fig.docx]

**
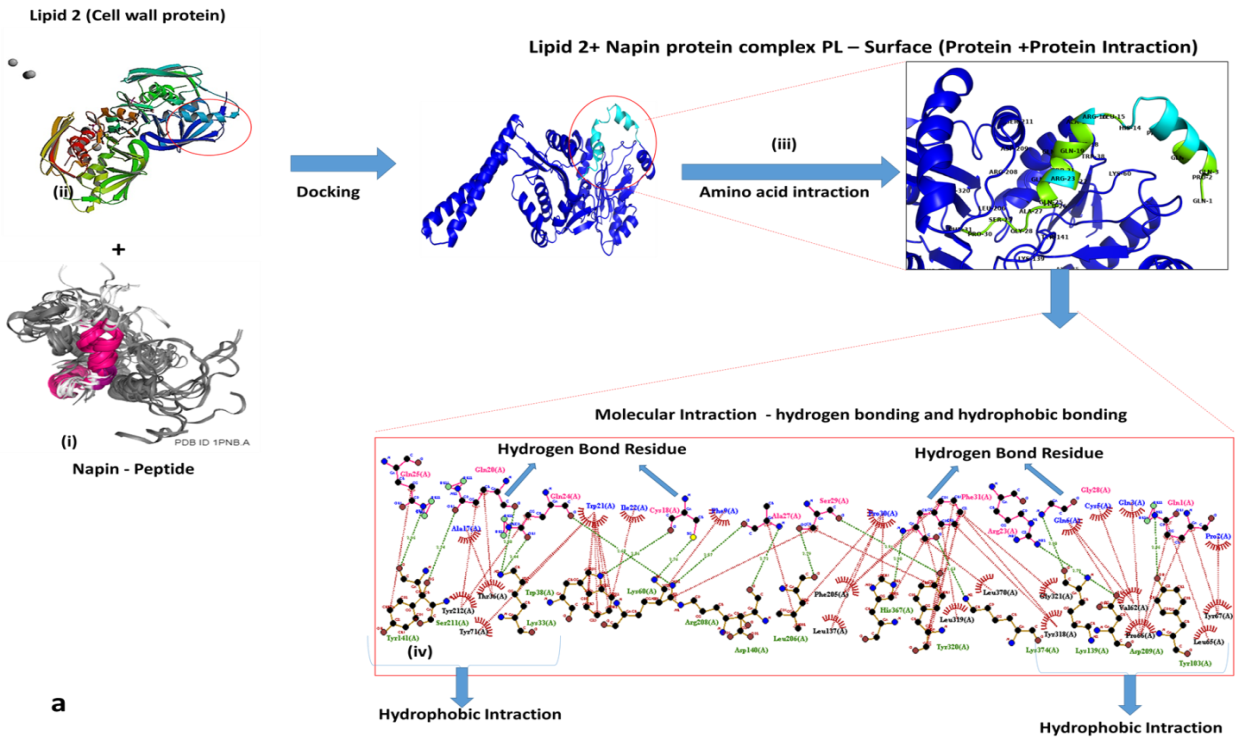
**

| **Napin_lipid2.** | **Napin** | **Lipid2** | **Total** | **NPN-LIPID2** | **Total-MSA** |
| --- | --- | --- | --- | --- | --- |
|  | 2768.19 | 21112.46 | 23880.65 | 19727.05 | 4153.60 |

**Supplementary Figure 1.** **(a**) (**i**) The interaction analysis between napin (pink) and lipid 2 (blue) by molecular docking; (**ii**) the interaction model of complex napin and lipid 2−3D structure of protein complex; (**iii**) interfaces and key residues analysis; (**iv**) Ligplot representation showing hydrogen and hydrophobic interactions between pediocin and lipid 2 complex and surface area assessment table for best docked models.
